# Prevalence and co-incidence of geriatric syndromes according to the ECOG performance status in older cancer patients

**DOI:** 10.3389/fmed.2024.1331246

**Published:** 2024-02-19

**Authors:** Atakan Topcu, Ayse Irem Yasin, Mehmet Besiroglu, Zehra Sucuoglu Isleyen, Zeynep Alaca Topcu, Melih Simsek, Haci Mehmet Turk, Mesut Seker, Pinar Soysal

**Affiliations:** ^1^Bezmialem Vakif University Hospital, İstanbul, Türkiye; ^2^Istanbul Medeniyet University Göztepe Prof Dr Süleyman Yalçın City Hospital, İstanbul, Türkiye

**Keywords:** cancer, ECOG performance status, geriatric syndrome, prevalence, frailty

## Abstract

**Background:**

Geriatric syndromes may be more common in older cancer patients than in those without cancer. Geriatric syndromes can cause poor clinical outcomes. The Eastern Cooperative Oncology Group Performance Status (ECOG-PS) is often used as a clinically reported functional status score in oncology practice.

**Methods:**

Our study was designed as a cross-sectional study and included 218 older cancer patients. This study aimed to determine the prevalence and relationship of geriatric syndromes according to the ECOG-PS in older cancer patients.

**Results:**

The mean age of 218 participants was 73.0 ± 5.6 years, with 47.7% being women and 52.3% men in our study. ECOG-PS 0, 1, and 2 groups contained 51, 39, and 10% of the patients, respectively. The mean number of geriatric syndromes in the ECOG 0, 1, and 2 groups was 2.3 ± 2.2, 4.3 ± 2.4, and 5.7 ± 2.1, respectively (*p* < 0.001). After adjusting for age and sex, it was determined that dynapenia was 2.9 times, probable sarcopenia was 3.5 times, frailty was 4.2 times, depression was 2.6 times, malnutrition was 3.3 times, insomnia 2 was.2 times, falls was 2.5 times, and the risk of falling (TUG) was 2.4 times more likely in those with ECOG-PS 1 compared to those with ECOG-PS 0. In addition, it was found that dynapenia was 6 times, probable sarcopenia was 6.8 times, frailty was 10.8 times, depression was 3.3 times, malnutrition was 6.3 times, the risk of falling (Tinnetti Balance) was 28 times, and the risk of falling (TUG) was 13.6 times more likely in those with ECOG-PS 2 compared to those with ECOG-PS 0.

**Conclusion:**

Our study found that the prevalence of geriatric syndromes increased as the ECOG-PS increased. Geriatric syndromes and their co-incidence were common in older cancer patients, even in normal performance status. Oncologists should incorporate geriatric syndromes into the decision-making process of cancer treatment to maximize the impact on clinical outcomes in older patients with cancer.

## Introduction

Cancer is detected more often with aging, as age represents the most potent non-modifiable risk factor for developing cancer (1). Aging is a biological process associated with a decrease in the capacity of all organ functions, and this causes individuals to be more vulnerable (2). While older cancer patients struggle with the problems caused by cancer and its treatments, they also have to fight the natural burden of aging. In clinical practice, oncologists frequently use the Eastern Cooperative Oncology Group Performance Status (ECOG-PS), which describes the patient’s baseline functional and frailty status. However, ECOG-PS may have a poor correlation with comprehensive geriatric assessment (CGA), and its usefulness in older adults with cancer is often questioned (3, 4). Although ECOG-PS is widely used by oncologists for frailty assessment, it is known that frailty is not the only issue, and geriatric syndromes can impair the quality of life and decrease the overall survival of cancer patients (5).

Assessment of older cancer patients can be more difficult for clinicians because age and tumor characteristics alone may not capture this patient group’s functional and frailty status (6, 7). Functional status can vary significantly among older cancer patients of similar chronological age. Therefore, it has been shown that CGA can add fundamental aid for the evaluation of functional assessment and treatment planning of older cancer patients, including those with a good PS (4, 8, 9).

Geriatric syndromes can be more common in older cancer patients than in those without cancer. For example, malnutrition may be common in older cancer patients because of nausea and vomiting that may develop due to chemotherapy (10). In addition, drugs that can be used to control symptoms and side effects such as nausea, fatigue, and pain may increase the frequency of polypharmacy (11). While geriatric syndromes are multifactorial, the presence of cancer also contributes to the development of these syndromes (2, 12). These syndromes can cause poor clinical outcomes, including increased morbidity and mortality. Therefore, identifying patients with geriatric syndromes is crucial in providing personalized care for older cancer patients (2, 5, 13, 14).

Geriatric syndromes such as malnutrition, frailty, fear of falling, falls, depression, insomnia, excessive daytime sleepiness, dynapenia, and sarcopenia are common in older patients. Although each geriatric syndrome has been evaluated separately in older cancer patients, there are a few studies examining all geriatric syndromes together and categorizing them based on their performance status (15, 16). Additionally, our real-life research also involves various geriatric syndromes, such as urinary incontinence, excessive daytime sleepiness, insomnia, dynapenia, and probable sarcopenia. This study aimed to determine the prevalence and relationship of geriatric syndromes according to the ECOG performance status in older cancer patients and to increase awareness of this issue.

## Methods

### Patients

Cancer patients of age ≥ 65 years were recruited from an ongoing longitudinal cohort treated at the University of Bezmialem Vakif University Oncology outpatient clinic from October 2020 to October 2022. Our study included 218 older cancer patients. The patients with Eastern Cooperative Oncology Group Performance Status ≥3, those under the age of 65 years, those with dementia or delirium, those with vision and hearing impairments that control understanding commands during the evaluation, and those with uncontrolled comorbid disorders were excluded. The study was designed as a cross-sectional study and was approved by the local ethics committee. Each participant provided informed consent before participating in the study.

### Oncological status

The cancer types of the participants were categorized into five subgroups: lung, breast, gastrointestinal system, urogenital system, and others. The current stages of the patients were divided into four subgroups, as stage 1, 2, 3, and 4, and they were also evaluated as metastatic and non-metastatic diseases. In addition, we noted whether the patients received active chemotherapy for their disease.

### The evaluation of functional status

The ECOG-PS is the most generally used clinically reported functional status score in oncology practice (17). Clinicians prefer one statement suitably defining the patient’s level of physical activity (ranging from 0 to 4). The scores were defined as 0 being fully active, 1 being restricted in strenuous activity, and 2 indicating being capable of all self-care but an inability to carry out any work activities, up and about more than 50% of waking hours. Scores of 3 and above indicate severe disability. Patients with ECOG-PS ≥3 have a poor prognosis and were excluded from our study.

### Comprehensive geriatric assessment

Sex, age, BMI, mean upper arm and calf circumference, smoking, number of drugs, cancer type and stage, chemotherapy status, cancer treatment history, history of falling, comorbidities including diabetes mellitus, hyperlipidemia, hypertension, coronary artery disease, cerebrovascular events, chronic obstructive pulmonary disease, and Parkinson’s disease were recorded. The following geriatric syndromes were assessed in interviews with the patient and the caregiver. The evaluation of each geriatric syndrome mentioned below has been detailed in our previous studies (13).

(1) Dynapenia: Dominant hand grip strength measured by a handgrip dynamometer less than 16 kg in women and less than 27 kg in men was defined as dynapenia (18).
(2) Probable sarcopenia: If SARC-*F* ≥ 4 + dynapenia was low according to the defined cutoff (19).
(3) Frailty: A modified Fried physical frailty scale was used to evaluate frailty, which was defined according to the physical model and the presence of three or more of the following criteria: weight loss, exhaustion, low physical activity, slowness, and weakness. Those with 0 were considered normal, while those with 1–2 criteria were considered pre-frailty and those with ≥3 were considered frailty (13).
(4) Depression: Yesavage Geriatric Depression Scale-15 (GDS-15) score ≥ 5 (20).
(5) Malnutrition: MNA long form scores <17 points were considered as having malnutrition. It was specified as 17–23.5 for at-risk malnutrition and ≥ 24 for normal nutritional status (20).
(6) Insomnia: Insomnia Severity Index score ≥ 8 (20).
(7) Excessive daytime sleepiness: Epworth Sleepiness Scale score ≥ 11 (20).
(8) Polypharmacy: The concurrent use of five or more drugs (20).
(9) Appetite assessment: <28 was defined as losing appetite according to the Council on Nutrition Appetite Questionnaire (CNAQ) (21).
(10) Falls: The patient’s fall was considered positive, except for slipping on the wet floor in the previous year (13).
(11) Risk of falling: A value of <19 was accepted according to the Tinetti Balance and Gait Test and ≥ 13.5 s according to the timed up and go (TUG) test (22, 23).

### Statistical analysis

Quantitative variables are defined as mean ± standard deviation, and qualitative variables are given as numbers and proportions. A chi-squared test was utilized for comparing qualitative measures between groups. Comparisons of continuous variables between the groups were achieved using the Kruskal–Wallis test. Significant differences were compared by the Bonferroni-corrected Mann–Whitney test. Factors associated with each geriatric syndrome were assessed using univariate logistic regression analysis. The model, therefore, was adjusted for age and sex. Results are described as odds ratio (OR) with a 95% confidence interval. IBM SPSS Statistics for Windows, Version 28.0. (Armonk, NY: IBM Corp.) was used for analysis. The level of significance was determined at a *p-*value of <0.05.

## Results

**Figure 1:**
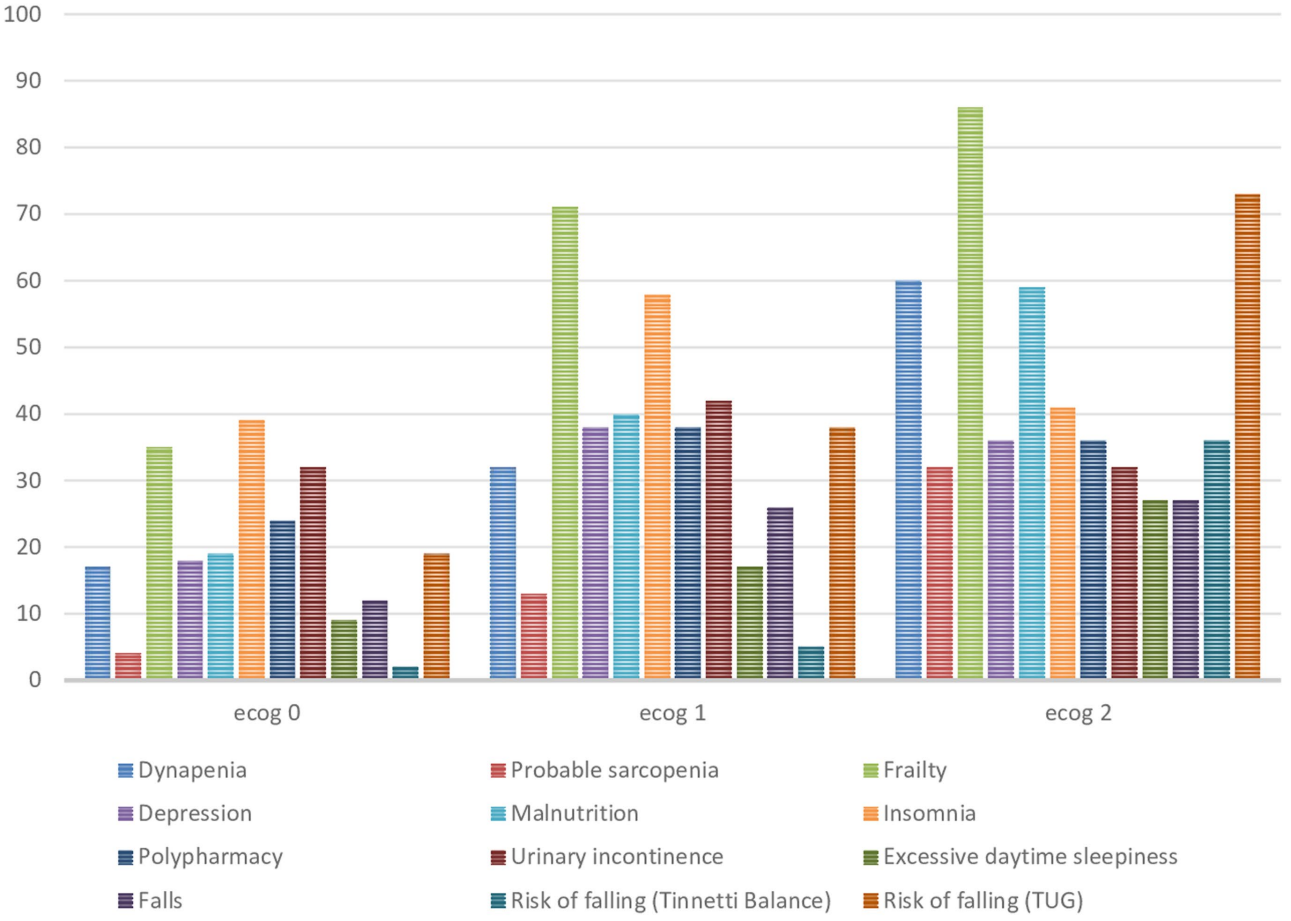
Frequency of geriatric syndromes based on the ECOG-PS groups (%).

**Figure 2:**
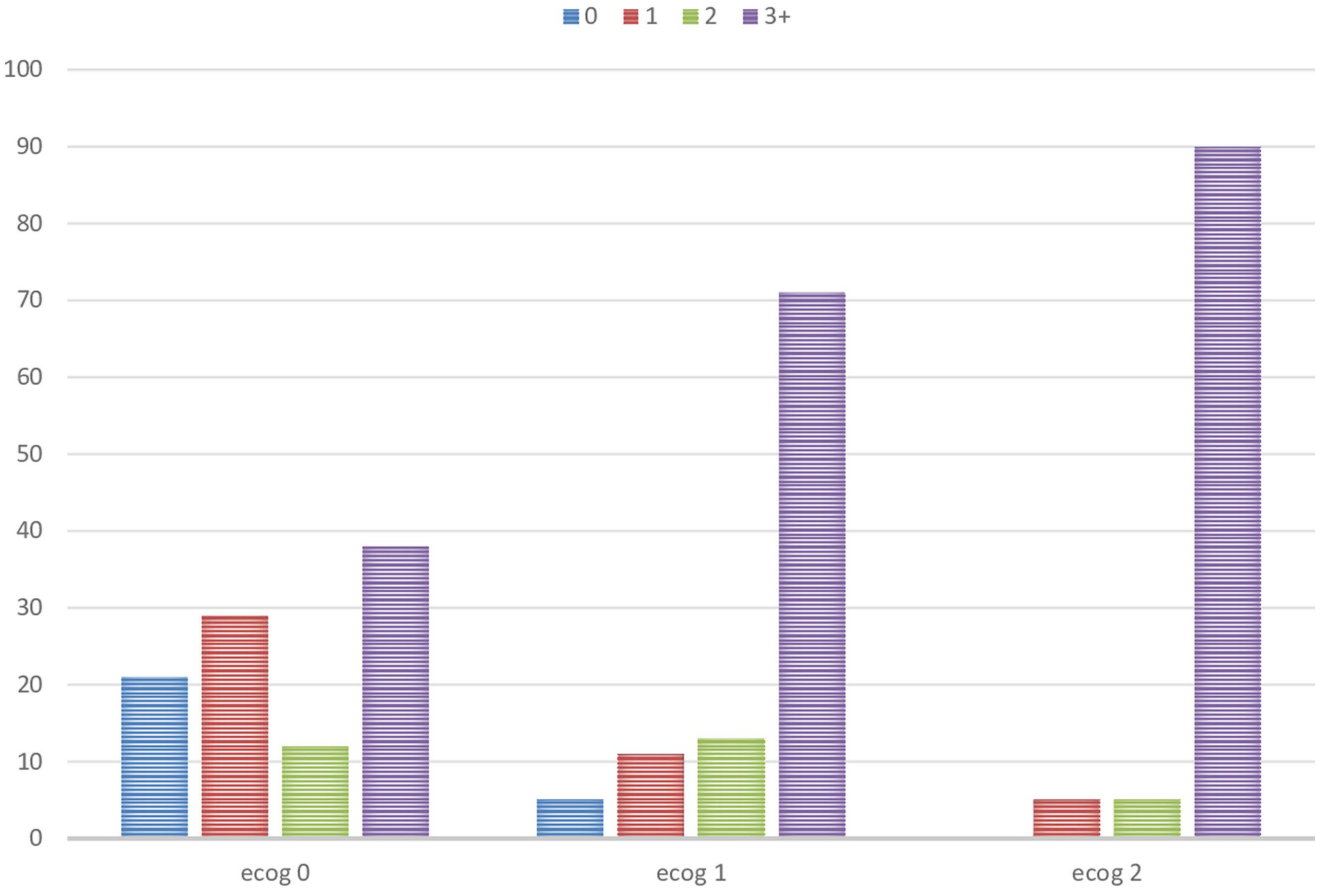
Total number of geriatric syndromes based on the ECOG-PS groups (%).

The mean age of 218 participants was 73.0 ± 5.6 years, with 104 (47.7%) women and 114 (52.3%) men in our study. The ECOG-PS 0, 1, and 2 groups contained 51, 39, and 10% of the patients, respectively. The mean age of the patients (*p* < 0.001) and the prevalence of the metastatic stage (*p* < 0.001) increased as the ECOG-PS score increased. Gastrointestinal cancers (39.9%) were the most common cancers in our study. The mean number of geriatric syndromes in the ECOG 0, 1, and 2 groups was 2.3 ± 2.2, 4.3 ± 2.4, and 5.7 ± 2.1, respectively (*p* < 0.001). The prevalence of geriatric syndromes increased as the ECOG-PS score increased. The prevalence of frailty, malnutrition, EDS, depression, dynapenia, and probable sarcopenia increased as the ECOG-PS score increased in our study. While the mean of the handgrip, MNA, and CNAQ of the patients decreased as the ECOG-PS score increased, the mean of the falls, risk of falling (TUG and Tinetti Balance), GDS, SARC-F, ISI, and Epworth scores increased. However, upper arm circumference, calf circumference, urinary incontinence, and loss of appetite were not different in the ECOG-PS groups. The characteristics of the patients and all geriatric syndrome comparisons based on the ECOG-PS category are shown in Table 1. The frequency of the geriatric syndromes increased in higher ECOG-PS groups (Figure 1). Among the geriatric syndromes, insomnia had the highest incidence in the ECOG-PS 0 group (39%), while frailty had the highest incidence in the ECOG-PS 1 group (71%), and the ECOG-PS 2 group (86%). The total number of geriatric syndromes based on the ECOG-PS groups is shown in Figure 2. In the ECOG-PS 0 group, 21% of the had 0 geriatric syndrome, 29% of the patients had 1 geriatric syndrome, 12% of the patients had 2 geriatric syndromes, and 38% of the patients had 3 or more geriatric syndromes. In addition, 5% of the patients had 0 geriatric syndrome, 11% of the patients had 1 geriatric syndrome, 13% of the patients had 2 geriatric syndromes, and 71% of the patients had 3 or more geriatric syndromes in the ECOG-PS 1 group. However, there was no patient without a geriatric syndrome in the ECOG-PS 2 group. Furthermore, 5% of the patients had one geriatric syndrome, another 5% of the patients had two geriatric syndromes, and 90% of the patients had three or more geriatric syndromes. Among the patients with an ECOG-PS of 0 or 1, we analyzed the OR of an ECOG-PS of 1 versus an ECOG-PS of 0 for each geriatric syndrome (Table 2). In addition, we also analyzed the OR of ECOG-PS 2 vs. ECOG-PS 0 for each geriatric syndrome (Table 3). After adjusting for age and sex, the ORs were significantly higher in the ECOC-PS 1 group compared to the ECOC-PS 0 group, as follows: dynapenia OR: 2.9, probable sarcopenia OR: 3.54, frailty OR: 4.2, depression OR: 2.68, malnutrition OR: 3.38, insomnia OR: 2.21, falls OR: 2.52, and risk of falling (TUG) OR: 2.41. There was no difference in terms of polypharmacy, urinary incontinence, excessive daytime sleepiness, and risk of falling (Tinetti Balance) in the ECOG-PS 0 and 1 groups. Moreover, the OR rates were significantly determined in the ECOC-PS 2 group compared to the ECOC-PS 0 group as follows: dynapenia OR: 6.03, probable sarcopenia OR: 6.85, frailty OR: 10.86, depression OR: 3.34, malnutrition OR: 6.32, risk of falling (Tinnetti Balance) OR: 28.01 times, and risk of falling (TUG) OR: 13.66. There was no difference in terms of insomnia, polypharmacy, urinary incontinence, excessive daytime sleepiness, and falls in the ECOG-PS 0 and 2 groups. We also conducted an analysis adjusted for age, sex, polypharmacy, and comorbidity, both for the group with ECOG-PS 1 vs. ECOG-PS 0 (Table 4) and for the group with ECOG-PS 2 vs. ECOG-PS 0 (Table 5).

**Table 1:** Characteristics of all patients and patient groups based on the ECOG-PS category.

Variables	All patients (*n* = 218)	ECOG-PS 0 (*n* = 111)	ECOG-PS 1 (*n* = 85)	ECOG-PS 2 (*n* = 22)	*p-*value
Age	73.0 ± 5.6	72.1 ± 4.7	73.0 ± 5.8	77.8 ± 7.0	<0.001
Sex					
Female	104 (47.7%)	45.8%	52.9%	36.4%	0.332
Male	114 (52.3%)	54.1%	47.1%	63.6%	
Smoking					
Yes	129 (59.2%)	63.1%	55.3%	54.5%	0.492
No	89 (40.8%)	36.9%	44.7%	45.5%	
Number of drugs	3.2 ± 2.7	2.9 ± 2.5	3.4 ± 2.8	3.8 ± 2.8	0.215
Polypharmacy (≥5)					
Yes	67 (30.7%)	24.3%	37.6%	36.4%	0.112
No	151 (69.3%)	75.7%	62.4%	63.6%	
Education, years	5.2 ± 3.7	5.6 ± 3.8	4.9 ± 3.9	4.2 ± 2.3	0.171
Stage					
Stage-1	13 (6.0%)	8.1%	3.5%	4.5%	0.008
Stage-2	42 (19.3%)	26.1%	14.1%	4.5%	
Stage-3	63 (28.9%)	32.4%	25.9%	22.7%	
Stage-4	100 (45.9%)	33.3%	54.5%	68.2%	
Stage category					
Non-metastatic	118 (54.1%)	66.7%	43.5%	31.8%	<0.001
Metastatic	100 (45.9%)	33.3%	56.5%	68.2%	
Diagnosis					
Lung cancer	35 (16.1%)	14.4%	14.5%	22.7%	0.004
Breast cancer	52 (23.9%)	22.5%	28.2%	13.6%	
Gastrointestinal cancer	87 (39.9%)	50.5%	29.4%	27.3%	
Urogenitale cancer	37 (17.0%)	10.8%	23.5%	22.7%	
Other cancer	7 (3.2%)	1.8%	2.4%	13.6%	
Active chemotherapy					
Yes	76 (34.9%)	28.8%	38.8%	50.0%	0.101
No	142 (65.1%)	71.2%	61.2%	50.0%	
Previously chemotherapy					
Yes	149 (68.3%)	69.4%	71.8%	50.0%	0.140
No	69 (31.7%)	30.6%	28.2%	50.0%	
Previous surgery					
Yes	144 (66.1%)	75.7%	60.0%	40.9%	0.002
No	74 (33.9%)	24.3%	40.0%	59.1%	
BMI	27.7 ± 4.9	27.8 ± 4.7	27.8 ± 5.1	27.0 ± 5.1	0.775
Diabetes mellitus					
Yes	69 (31.7%)	32.4%	30.6%	31.8%	0.963
No	149 (68.3%)	67.6%	69.4%	68.2%	
Hypertension					
Yes	125 (57.3%)	56.8%	60.0%	50.0%	0.689
No	93 (42.7%)	43.2%	40.0%	50.0%	
Hyperlipidemia					
Yes	26 (11.9%)	11.7%	11.8%	13.6%	0.966
No	192 (88.1%)	88.3%	88.2%	84.6%	
Coronary artery disease					
Yes	37 (17%)	16.2%	15.3%	27.3%	0.392
No	181 (83%)	83.8%	84.7%	72.7%	
Cerebrovascular events					
Yes	6 (2.8%)	0.9%	4.7%	4.5%	0.235
No	212 (97.2%)	99.1%	95.3%	95.5%	
COPD					
Yes	10 (4.6%)	4.5%	3.5%	9.1%	0.538
No	208 (95.4%)	95.5%	96.5%	90.9%	
Parkinson’s disease					
Yes	3 (1.4%)	0%	3.5%	0%	0.093
No	215 (98.6%)	100%	96.5%	100%	
SARC-F	1.9 ± 2.4	0.9 ± 1.5	2.4 ± 2.5	4.9 ± 2.9	<0.001
SARC-F category					
≥4	42 (19.3%)	8.1%	23.5%	59.1%	<0.001
<4	176 (80.7%)	91.9%	76.5%	40.9%	
MNA score	24.3 ± 4.1	25.8 ± 3.1	23.3 ± 3.8	20.4 ± 5.6	<0.001
Nutrition status					
Normal nutrition	121 (64%)	78.8%	51.4%	35%	<0.001
Malnutrition	68 (36%)	21.2%	48.6%	65%	
Malnutrition category					
Normal nutrition	121 (64%)	78.8%	51.4%	35%	<0.001
Malnutrition risk	57 (30.2%)	21.2%	40%	40%	
Malnutrition	11 (5.8%)	0%	8.6%	25%	
Upper arm circumference	27.3 ± 4.4	27.8 ± 4.5	27.2 ± 4.1	25.4 ± 4.9	0.110
Calf circumference	35.7 ± 4.8	36 ± 4.6	35.9 ± 5	33.3 ± 4.9	0.094
Incontinence					
Yes	79 (36.2%)	32.4%	42.4%	31.8%	0.323
No	139 (63.8%)	67.6%	57.6%	68.2%	
Tinetti Gait and Balance Test	25.9 ± 4	27.4 ± 1.9	25.6 ± 3.5	19.8 ± 6.8	<0.001
Risk of falling					
Yes (Tinetti Balance <19)	14 (6.5%)	1.8%	4.9%	36.4%	<0.001
No (Tinetti Balance ≥19)	201 (93.5%)	98.2%	95.1%	63.6%	
Falls					
Yes	41 (18.8%)	11.7%	25.9%	27.3%	0.024
No	177 (81.2%)	88.3%	74.1%	72.7%	
The time up and go (TUG)	13.5 ± 7.9	11.4 ± 3.2	13.5 ± 4.8	24.2 ± 19.4	<0.001
Risk of falling					
Yes (TUG ≥13.5)	69 (33.7%)	20.2%	39.5%	80%	<0.001
No (TUG <13.5)	136 (66.3%)	79.8%	60.5%	20%	
Handgrip (highest)	25.1 ± 9.8	27.4 ± 9.5	23.6 ± 9.7	17.9 ± 6.6	<0.001
Dynapenia					
Yes	59 (32.4%)	19%	42.2%	72.2%	<0.001
No	123 (67.6%)	81%	57.8%	27.8%	
Probable sarcopenia					
Yes	22 (10.1%)	3.6%	12.9%	31.8%	<0.001
No	196 (89.9%)	96.4%	87.1%	68.2%	
GDS-15	3.4 ± 3.1	2.5 ± 2.7	4.1 ± 3	5.1 ± 4.1	<0.001
Depression					
Yes (GDS-15 ≥ 5)	60 (27.6%)	18%	37.6%	38.1%	0.005
No (GDS-15 < 5)	157 (72.4%)	82%	62.4%	61.9%	
Epworth	4.7 ± 4.7	3.9 ± 4	5.2 ± 5.1	7.1 ± 4.9	0.005
Excessive daytime sleepiness					
Yes (Epworth ≥11)	30 (13.9%)	9.1%	16.5%	28.6%	0.041
No (Epworth <11)	186 (86.1%)	90.9%	83.5%	71.4%	
ISI	9.1 ± 8.2	7.4 ± 7.3	10.8 ± 8.4	11.2 ± 10.2	0.007
Insomnia					
Yes (ISI ≥8)	101 (46.3%)	38.7%	57.6%	40.9%	0.027
No (ISI <8)	117 (53.7%)	61.3%	42.4%	59.1%	
Frailty score	1.3 ± 1.5	0.7 ± 1.1	1.7 ± 1.5	3.5 ± 1.6	<0.001
Frailty					
Yes	118 (54.4%)	35.5%	70.6%	86.4%	<0.001
No	99 (45.6%)	64.5%	29.4%	13.6%	
Frailty category					
Frailty	51 (23.5%)	8.2%	30.6%	73.8%	<0.001
Pre-frailty	67 (30.9%)	27.3%	40%	13.6%	
Normal	99 (45.6%)	64.5%	29.4%	13.6%	
CNAQ	28.1 ± 5.1	29.1 ± 4.6	27.8 ± 4.5	24.4 ± 7.7	<0.001
Losing appetite					
Yes (CNAQ <28)	75 (34.7%)	28.2%	40%	47.6%	0.097
No (CNAQ ≥28)	141 (65.3%)	71.8%	60%	52.4%	
Geriatric syndromes	3.4 ± 2.6	2.3 ± 2.2	4.3 ± 2.4	5.7 ± 2.1	<0.001

**Table 2:** Among patients with ECOG-PS 0 or 1, odds ratio of ECOG-PS 1 vs. ECOG-PS 0 for each geriatric syndrome.

	Univariate			Age- and sex-matched		
	OR	%95CI	*p*	OR	%95CI	*p*
Dynapenia	3.11	1.54–6.29	0.002	2.90	1.39–6.07	0.004
Probable sarcopenia	3.98	1.22–12.97	0.022	3.54	1.06–11.83	0.040
Frailty	4.37	2.38–8.03	<0.001	4.20	2.27–7.76	<0.001
Depression	2.75	1.43–5.28	0.002	2.68	1.36–5.29	0.004
Malnutrition	3.51	1.79–6.87	<0.001	3.38	1.71–6.68	<0.001
Insomnia	2.15	1.21–3.83	0.009	2.21	1.22–3.98	0.009
Polypharmacy	1.88	1.01–3.48	0.045	1.74	0.92–3.28	0.091
Urinary incontinence	1.53	0.85–2.75	0.154	1.45	0.80–2.63	0.218
Excessive daytime sleepiness	1.97	0.83–4.69	0.125	1.93	0.81–4.63	0.140
Falls	2.63	1.24–5.60	0.012	2.52	1.18–5.40	0.017
Risk of falling (Tinnetti Balance)	2.80	0.50–15.64	0.242	2.06	0.34–12.42	0.431
Risk of falling (TUG)	2.58	1.34–4.96	0.004	2.41	1.21–4.78	0.012

**Table 3:** Among patients with ECOG-PS 0 or 2, odds ratio of ECOG-PS 2 vs. ECOG-PS 0 for each geriatric syndrome.

	Univariate			Age- and sex-matched		
	OR	%95CI	*p*	OR	%95CI	*p*
Dynapenia	11.08	3.52–34.87	<0.001	6.03	1.71–21.25	0.005
Probable sarcopenia	12.48	3.26–47.78	<0.001	6.85	1.53–30.69	0.012
Frailty	11.53	3.21–41.42	<0.001	10.86	2.80–42.19	0.001
Depression	2.80	1.03–7.65	0.045	3.34	1.07–10.37	0.037
Malnutrition	6.90	2.44–19.47	<0.001	6.32	2.00–19.94	0.002
Insomnia	1.10	0.43–2.78	0.849	1.40	0.50–3.95	0.521
Polypharmacy	1.78	0.67–4.69	0.245	1.50	0.50–4.52	0.468
Urinary incontinence	0.97	0.36–2.59	0.955	0.71	0.24–2.16	0.551
Excessive daytime sleepiness	4.00	1.27–12.61	0.018	3.03	0.82–11.19	0.096
Falls	2.83	0.94–8.51	0.065	1.92	0.54–6.78	0.311
Risk of falling (Tinnetti Balance)	31.14	6.00–161.57	<0.001	28.01	4.68–167,59	<0.001
Risk of falling (TUG)	15.81	4.78–52.26	<0.001	13.66	3.69–50.65	<0.001

**Table 4:** Among patients with ECOG-PS 0 or 1, the odds ratio of ECOG-PS 1 vs. ECOG-PS 0 for each geriatric syndrome (age, sex, polypharmacy, and comorbidity-matched).

	Univariate			Age, sex, polypharmacy, and comorbidity-matched		
	OR	%95CI	*p*	OR	%95CI	*p*
Dynapenia	3.11	1.54–6.29	0.002	3.01	1.41–6.40	0.004
Probable sarcopenia	3.98	1.22–12.97	0.022	4.22	1.23–14.49	0.022
Frailty	4.37	2.38–8.03	<0.001	4.54	2.39–8.62	<0.001
Depression	2.75	1.43–5.28	0.002	2.62	1.31–5.23	0.007
Malnutrition	3.51	1.79–6.87	<0.001	3.52	1.73–7.19	0.001
Insomnia	2.15	1.21–3.83	0.009	2.34	1.27–4.31	0.006
Polypharmacy	1.88	1.01–3.48	0.045	2.29	1.12–4.69	0.024
Urinary incontinence	1.53	0.85–2.75	0.154	1.44	0.78–2.64	0.246
Excessive daytime sleepiness	1.97	0.83–4.69	0.125	2.14	0.87–5.25	0.096
Falls	2.63	1.24–5.60	0.012	2.52	1.45–5.52	0.021
Risk of falling (Tinnetti Balance)	2.80	0.50–15.64	0.242	2.14	0.34–13.29	0.415
Risk of falling (TUG)	2.58	1.34–4.96	0.004	2.49	1.24–5.00	0.011

**Table 5:** Among patients with ECOG-PS 0 or 2, the odds ratio of ECOG-PS 2 vs. ECOG-PS 0 for each geriatric syndrome (age, sex, polypharmacy, and, comorbidity-matched).

	Univariate			Age, sex, polypharmacy, and comorbidity-matched		
	OR	%95CI	*p*	OR	%95CI	*p*
Dynapenia	3.11	1.54–6.29	0.002	5.73	1.59–20.59	0.008
Probable sarcopenia	3.98	1.22–12.97	0.022	6.67	1.48–30.07	0.014
Frailty	4.37	2.38–8.03	<0.001	11.05	2.79–43.71	0.001
Depression	2.75	1.43–5.28	0.002	3.32	1.06–10.41	0.040
Malnutrition	3.51	1.79–6.87	<0.001	9.08	2.47–33.43	0.001
Insomnia	2.15	1.21–3.83	0.009	1.37	0.48–3.93	0.554
Polypharmacy	1.88	1.01–3.48	0.045	1.52	0.46–5.07	0.492
Urinary incontinence	1.53	0.85–2.75	0.154	0.67	0.22–2.07	0.487
Excessive daytime sleepiness	1.97	0.83–4.69	0.125	3.34	0.87–12.86	0.079
Falls	2.63	1.24–5.60	0.012	1.97	0.55–7.03	0.296
Risk of falling (Tinnetti Balance)	2.80	0.50–15.64	0.242	27.85	4.56–170.01	<0.001
Risk of falling (TUG)	2.58	1.34–4.96	0.004	13.62	3.62–51.20	<0.001

## Discussion

In our study, the frequency of geriatric syndromes increased as ECOG-PS increased. Frailty was the most common geriatric syndrome in both the ECOG-PS 1 and ECOG-PS 2 groups. Moreover, in the ECOG-PS 0 group, it was the second most common geriatric syndrome after insomnia. Most patients had three or more geriatric syndromes in the ECOG-PS 1 and ECOG-PS 2 groups. In addition, in the ECOG-PS 2 group, all patients had at least one geriatric syndrome. The mean age of the patients and the prevalence of the metastatic stage increased as the ECOG-PS score increased. After adjusting for age and sex, it was determined that dynapenia was 2.9 times, probable sarcopenia was 3.5 times, frailty was 4.2 times, depression was 2.6 times, malnutrition was 3.3 times, insomnia was 2.2 times, falls was 2.5 times, and the risk of falling (TUG) was 2.4 times more likely in those with ECOG-PS 1 compared to those with ECOG-PS 0. Frailty had the highest OR. In addition, it was found that dynapenia was 6 times, probable sarcopenia was 6.8 times, frailty was 10.8 times, depression was 3.3 times, malnutrition was 6.3 times, and the risk of falling (Tinnetti Balance) was 28 times, and the risk of falling (TUG) was 13.6 times more likely in those with ECOG-PS 2 compared to those with ECOG-PS 0. The risk of falling (Tinnetti Balance) had reached the highest OR. In addition, there was no difference in the ORs between both groups (ECOG-PS 1 vs. 0 and ECOG-PS 2 vs. 0) concerning polypharmacy, urinary incontinence, and excessive daytime sleepiness in our study.

The ECOG-PS provides limited information about the patient and may be insufficient for the treatment of older cancer patients (24). An important review determined that CGA could play an effect modifier role for the results of oncological treatment and could give substantial information in addition to chronological age and performance score (25). Recent studies have underlined the importance of CGA in older cancer patients to improve treatment planning (12, 26). In a geriatric study involving multiple myeloma patients, they found that at least two geriatric assessment-identified deficits were observed in 41% of those with a normal performance score with Karnofsky Performance Status (KPS), which is another validated score for functional evaluation in cancer patients (27). In a study that included older cancer patients with normal performance status, they found at least one geriatric syndrome in 69% of the patients. They also noted that the CGA identified substantial deficits that could affect poor clinical outcomes even in normal performance status (3). Moreover, this study also pointed out that potentially modifiable deficits should be assessed with interventions in older cancer patients with normal performance scores, which is consistent with the recent studies (4, 14). In our study, 79% of the patients with normal performance status (ECOG-PS 0) had at least one geriatric syndrome. In addition, the ECOG-PS score was significantly correlated with the frequency of geriatric syndromes in the present study. Moreover, some geriatric syndromes were significantly more common in the ECOG-PS 2 group compared to the ECOG-PS 0 group or in the ECOG-PS 1 group compared to the ECOG-PS 0 group. Although ECOG-PS could play an important role in the management of cancer patients, our study found that 38% of the patients in the ECOG-PS 0 group had three or more geriatric syndromes. Moreover, even in the ECOG-PS 0 group, 39% of the patients had insomnia. In addition, in ECOG-PS 1 and 2 groups, most patients had three or more geriatric syndromes. In addition, a study in Japan found that geriatric assessment variables could predict systemic therapy toxicity in older cancer patients (28). They emphasize that ECOG-PS is frequently used in patients to predict treatment toxicity and mortality; however, it is not suitable or sufficient for predicting treatment outcomes in older cancer patients. In our study, more than two-thirds of the patients had a history of chemotherapy, and geriatric syndromes were common in patients with all ECOG-PS groups, even in the normal PS group. In our research, although the frequency of geriatric syndromes increased as ECOG-PS increased, the geriatric syndromes and co-incidence were common even in ECOG-PS 0 and 1 groups. Thus, the performance status reviews cannot possibly interrogate many parts of the CGA for older cancer patients (4). The comprehensive geriatric assessment provided crucial information on the functional assessment of older cancer patients, including patients with a good performance status. Considering the importance of these geriatric syndromes for the prognosis and clinical outcomes of older cancer patients, it is very crucial to detect and integrate these geriatric syndromes in the treatment assessment.

Depression is among the most common geriatric syndromes in patients with cancer, with prevalence estimates reaching as high as 25% (29). However, recognizing depression can be difficult and is often underdefined. In a study consisting mostly of gastrointestinal cancer patients, the prevalence of having at least one geriatric syndrome was 65.2%. Depression (30%) was the most common geriatric syndrome in this research (24). Similar to the findings of the above-mentioned study, gastrointestinal cancers were the most common cancer type in our study. In our research, at least one geriatric syndrome was present in 79% of the ECOG-PS 0 group, 95% of the ECOG-PS 1 group, and 100% of the ECOG-PS 2 group. Depression was also found at a similar rate (27.6%) in the present study. Moreover, in this study, which mostly included patients with good performance scores, urinary incontinence, one of the most important geriatric syndromes, was detected at very low rates. However, in our study, it was present in approximately one-third of the patients, and there was no difference between the ECOG-PS groups. Urinary incontinence is critical for older cancer patients because it is associated with many adverse outcomes, including depression, anxiety, poorer quality of life, higher mortality rates, falls, pressure ulcers, diabetes, arthritis, fecal incontinence, and frailty (30). A recent study showed that the risk of malnutrition is related to a poor prognosis in older cancer patients. Moreover, malnutrition can be associated with many geriatric syndromes (10). In our study, the risk of malnutrition had been considerably detected in older patients with cancer, and it was correlated with poor ECOG-PS status. Polypharmacy, an important geriatric syndrome, was shown to be predictive for chemotherapy toxicity and overall survival. It may be more common in older patients with cancer compared with the general geriatric population due to multiple factors (2). Polypharmacy was present in one-third of the patients in our study, and there was no difference between the ECOG-PS groups. In addition, sarcopenia may be a prognostic factor for treatment outcomes and the overall survival in older cancer patients. Therefore, oncologists should focus on the sarcopenia status of their patients when oncological treatment is planned (31). In our research, sarcopenia was detected more frequently in poor ECOG-PS groups. In addition, falls were prevalent among older cancer patients, and they were related to poor functional status and clinical outcomes (2, 32). In the present study, falls were more common in the ECOG 1 group than in the ECOG 0 group, and they were associated with other geriatric syndromes. In addition, sleeping disorders can also adversely affect patients’ quality of life and may even influence treatment outcomes in older patients with cancer (33). Insomnia was the most frequent geriatric syndrome in the ECOG-PS 0 group in our research. However, most of the patients had no excessive daytime sleepiness.

Frailty is defined as a result of vulnerability to the loss of reserve in response to stressors and a decline in physiological performance reserves and organ functions (34). It is a substantial predictor of poor treatment tolerance, treatment toxicities, decreased quality of life, and shorter survival in older patients with cancer (35, 36). It is often accepted as a frequent and underdiagnosed geriatric syndrome (37). In a Nordic study, it was found that the geriatric syndromes, including frailty and the ECOG-PS, showed prognostic value in older cancer patients (5). This study points out that frailty should be recognized at the initiation of the cancer treatment. Moreover, in a Norwegian study, they found that geriatric assessment was superior to oncologists’ clinical judgment in identifying frailty. In this study, 49% of the patients were frail. They found that only the geriatric assessment-frailty status could be a prognostic factor for survival (35). In our study, the prevalence and co-incidence of geriatric syndromes were common in all groups, including the ECOG-PS 0 group. In our research, frailty, the most common geriatric syndrome in the ECOG-PS 1 and 2 groups, was detected in 70 and 86% of the patients, respectively. In the ECOG-PS 0 group, frailty and insomnia were the most common geriatric syndromes, and they were detected in one-third of the patients. Moreover, frailty was 4.2 times (the highest ratio) more common in the ECOG-PS 1 group compared to the ECOG-PS 0 group. In addition, it was 10.8 times more common in the ECOG-PS 2 group compared to the ECOG-PS 0 group. It is shown that frailty can simply be identified with CGA. While oncologists usually use the ECOG-PS in clinical practice, it can have a poor correlation with the CGA, leading to questions about its usefulness in older cancer patients (3). Although using the ECOG-PS in clinical practice provides important prognostic information, the addition of CGA gives more crucial details for older frail patients with cancer (3, 35, 38).

Our study has some limitations. First, our research was a cross-sectional study. Our cancer sample was heterogeneous. Therefore, it may be difficult to generalize this conclusion to all cancer patients. In addition, we did not include all geriatric syndromes. Moreover, increasing the representation of ECOG-PS 2 patients by adding more participants could be effective in improving the study results. However, we state that our study provides substantial information for clinicians and their patients. It may be a reference for more comprehensive prospective studies planned in the future.

## Conclusion

In conclusion, our real-life study found that the prevalence of geriatric syndromes increased as ECOG-PS increased. Some geriatric syndromes were significantly more common as ECOG-PS increased. Moreover, geriatric syndromes and their co-incidence were common in older cancer patients, even in their normal performance status. Oncologists should incorporate geriatric syndromes into the decision-making process of cancer treatment to maximize the impact on clinical outcomes in older patients with cancer.

## Data availability statement

The original contributions presented in the study are included in the article/supplementary material, further inquiries can be directed to the corresponding author.

## Ethics statement

The studies involving humans were approved by the Clinical Research Ethics Committee of Bezmialem Vakif University. The studies were conducted in accordance with the local legislation and institutional requirements. The participants provided their written informed consent to participate in this study.

## Author contributions

AT: Data curation, Formal analysis, Investigation, Methodology, Project administration, Resources, Software, Writing – original draft, Writing – review & editing. AY: Data curation, Investigation, Writing – review & editing. MB: Investigation, Supervision, Writing – review & editing. ZS: Resources, Writing – review & editing. ZA: Data curation, Investigation, Writing – review & editing. MSi: Formal analysis, Writing – review & editing. HT: Visualization, Writing – review & editing. MSe: Methodology, Writing – review & editing. PS: Data curation, Investigation, Methodology, Project administration, Resources, Supervision, Writing – original draft, Writing – review & editing.
